# Influence of developmental stage and genotype on liver mRNA levels among wild, domesticated, and hybrid rainbow trout (*Oncorhynchus mykiss*)

**DOI:** 10.1186/1471-2164-14-673

**Published:** 2013-10-02

**Authors:** Samantha L White, Dionne Sakhrani, Roy G Danzmann, Robert H Devlin

**Affiliations:** 1 Fisheries and Oceans Canada, 4160 Marine Drive, West Vancouver, BC V7V 1N6, Canada; 2 Department of Integrative Biology, University of Guelph, Guelph, Ontario N1G 2W1, Canada

**Keywords:** Genomics, Domestication, Growth, Rainbow trout, Age-matched, Size-matched

## Abstract

**Background:**

Release of domesticated strains of fish into nature may pose a threat to wild populations with respect to their evolved genetic structure and fitness. Understanding alterations that have occurred in both physiology and genetics as a consequence of domestication can assist in evaluating the risks posed by introgression of domesticated genomes into wild genetic backgrounds, however the molecular causes of these consequences are currently poorly defined. The present study has examined levels of mRNA in fast-growing pure domesticated (D), slow-growing age-matched pure wild (Wa), slow-growing size-matched pure wild (Ws), and first generation hybrid cross (W/D) rainbow trout (*Oncorhynchus mykiss*) to investigate the influence of genotype (domesticated vs. wild, and their interactions in hybrids) and developmental stage (age- or size-matched animals) on genetic responses (i.e. dominant vs. recessive) and specific physiological pathways.

**Results:**

Highly significant differences in mRNA levels were found between domesticated and wild-type rainbow trout genotypes (321 mRNAs), with many mRNAs in the wild-domesticated hybrid progeny showing intermediate levels. Differences were also found between age-matched and size-matched wild-type trout groups (64 mRNAs), with unique mRNA differences for each of the wild-type groups when compared to domesticated trout (Wa: 114 mRNAs, Ws: 88 mRNAs), illustrating an influence of fish developmental stage affecting findings when used as comparator groups to other genotypes. Analysis of differentially expressed mRNAs (found for both wild-type trout to domesticated comparisons) among the genotypes indicates that 34.8% are regulated consistent with an additive genetic model, whereas 39.1% and 26.1% show a recessive or dominant mode of regulation, respectively. These molecular data are largely consistent with phenotypic data (growth and behavioural assessments) assessed in domesticated and wild trout strains.

**Conclusions:**

The present molecular data are concordant with domestication having clearly altered rainbow trout genomes and consequent phenotype from that of native wild populations. Although mainly additive responses were noted in hybrid progeny, the prevalence of dominant and non-additive responses reveals that introgression of domesticated and wild genotypes alters the type of genetic control of mRNA levels from that of wild-type, which may lead to disruption of gene regulation systems important for developing phenotypes for optimal fitness in nature. A clear influence of both fish age and size (developmental stage) on mRNA levels was also noted in this study, which highlights the importance of examining multiple control samples to provide a comprehensive understanding of changes observed between strains possessing differences in growth rate.

## Background

Domestication is a natural process that occurs in organisms subjected to rearing in animal husbandry, horticulture and aquaculture, adapting them to artificial environmental conditions which differ from those their progenitor wild strains evolved within. Domestication is often coupled with selective breeding to further accentuate desired phenotypic traits such as enhanced growth rate [1-3]. Salmonids (salmon, trout and their relatives) provide an ideal model for domestication due to variability between individual fish for many desirable phenotypic traits, and, unlike for many domesticated agricultural species, in most cases wild strains remain as comparators to the domesticated strains [4-8]. This difference in phenotype between wild and domesticated strains has been found by quantitative genetic studies to be determined mainly by additive genetic differences that accentuate phenotype in domesticated strains relative to the wild parental line [4-6,9,10]. Our understanding of both undirected and directed selection occurring during domestication is based mainly on observations of the phenotypic characteristics of the animal (e.g. body size at maturation), with knowledge regarding the specific underlying genetic and physiological features under selection still poorly understood. Improving our understanding of the molecular genetic basis of domestication will assist in identifying loci involved in control of phenotypic traits, both those desirable for the culture environment and those that may pose risks to wild strains. Indeed, of major concern to fish biologists is the intentional or unintentional release of domesticated fish stocks. Introgression of domesticated and wild genomes may result in hybrid progeny with reduced fitness for their environment [11-14]. However, a recent study by Skaala et al. has demonstrated improved survival of hybrid progeny (some year classes) in the wild [15]. To assist in addressing such concerns, it would be beneficial to understand genetic changes occurring during domestication as well as the effects of interacting genomes on gene regulatory processes. This knowledge would aid in the prediction of the detrimental outcomes of such crosses [12,16,17].

Inter-breeding between distinct populations can lead to different results depending on genetic and environmental factors. Hybridization of strains with low genetic variation and phenotypic range (e.g. from inbreeding or bottlenecks) may in theory yield hybrid progeny with desirable phenotypes through heterosis, where the offspring possess a more favourable phenotype than their parental strains [18]. Out-breeding depression can also occur where the off-spring express a non-favourable phenotype relative to parental strains, leading to reduced fitness of the hybrid progeny and the potential for negative impacts for wild stocks [16,19]. Phenotype-genotype relationships have been examined for traits such as survival, aggression, predator evasion, and feeding motivation [5,6,14,19-24], and for growth potential of wild-domesticated hybrid progeny in relation to their parental strains [4,5,7,8,25]. The overall findings of these studies suggested that domestication-induced traits are regulated mainly by additive genetic variation. Out-breeding with continually backcrossing of hybrid progeny into a wild genome may result in dilution of the domesticated-induced phenotype and a reversion to wild phenotype [26,27]. While these studies have focused mainly on the effect of domestication through quantification of the phenotypic trait in the hybrid relative to both parents, the advent of microarray technology has enabled exploration at the mRNA level. Research on the relationship between mRNA levels and phenotypic traits has been reported in mouse [28,29] and Drosophila *sp *[30-33]. However, several studies have also applied this technology to explore the genetic variation arisen through domestication in rainbow trout [34], Atlantic salmon [35-37], coho [38], brook charr [39,40] and lake whitefish [41] salmonid species.

The present study uses microarray technology to screen for differences in mRNA abundance levels between fast-growing pure domesticated, slow-growing pure wild, and wild-domesticated hybrid (intermediate growth) rainbow trout in liver tissue. Domesticated strains used within this study have been under selection for enhanced growth rate for greater than 30 years and show highly different growth rates relative to their wild comparators (leading to > 25-fold weight difference in domesticated compared to wild-type trout after 14 months), and are comparable to growth rates seen for growth hormone transgenic fish [42]. The main aims of this study were to (1) investigate changes in mRNA levels that have arisen through domestication, and relate these findings to growth and other physiological changes, (2) investigate the relationship among genotypes regarding their effects on mRNA levels, specifically that for wild-domesticated hybrids relative to parental strains, and (3) investigate the effect of developmental stage on mRNA levels by inclusion of both age-matched and size-matched wild-type reference groups. Previous studies of domestication [34,36,38] have tended to size and stage-match wild reference groups to domesticated populations in attempts to control for either developmental (body size, or stage) or environmental (rearing time) variance between groups due to size and stage differences. This study included both size-matched and age-matched wild groups as comparators.

## Methods

### Strains and fish culture

Strains of rainbow trout (*O. mykiss*) utilized in the present study were a wild strain (W; normally slow-growing) derived from Pennask Lake, British Columbia and a fast-growing domesticated (D) strain which has undergone selection for enhanced growth rate for over 30 years. The domesticated trout are derived from Campbell Lake Trout Farms (Little Fort, BC), a commercial strain used in aquaculture in British Columbia. In addition to the pure strains, wild-domesticated F_1_ hybrids (W/D) were also generated. These three genotypes of fish grow at very different rates, and thus to facilitate comparisons, wild strain trout were analyzed as age-matched (Wa) individuals and, using stock from the previous year, as individuals size-matched (Ws) to the D strain trout. Strains were generated and reared at the Fisheries and Oceans Canada Centre for Aquaculture and Environmental Research (CAER) in West Vancouver, BC. In June 2007, a series of crosses were performed to assess phenotypic and genetic relationships between the D and W strains. For the molecular analysis described here, a single cross was selected to minimize complications arising from allelic differences that may exist among individuals within each of the strains. Thus, groups were derived from 1) one female D trout crossed with a D male to generate pure domestic (D) trout, 2) the same D female was crossed with a W male to generate W/D hybrid progeny, and 3) to generate pure W trout, one W female was crossed with the same W male used to generate the W/D progeny. In the previous year (2006), W trout were selected from a large, randomly mated set of crosses from fish from the same wild source as for 2007 broods (Pennask Lake). All families (including size-matched wild-type trout) were reared individually under the same culture conditions in fresh aerated well water (10°C) at a density of less than 5 kg/m^3^ under simulated natural lighting and photoperiod (latitude 49˚13′). Fish were fed stage-appropriate artificial salmon diets (Sketting, Canada Ltd.) to satiation three times per day for the wild-type, domesticated and F_1_ hybrid groups. All experiments were approved by the DFO Pacific Regional Animal Care Committee and adhered to Guidelines of the Canadian Council for Animal Care.

### Sampling

**Figure 1:**
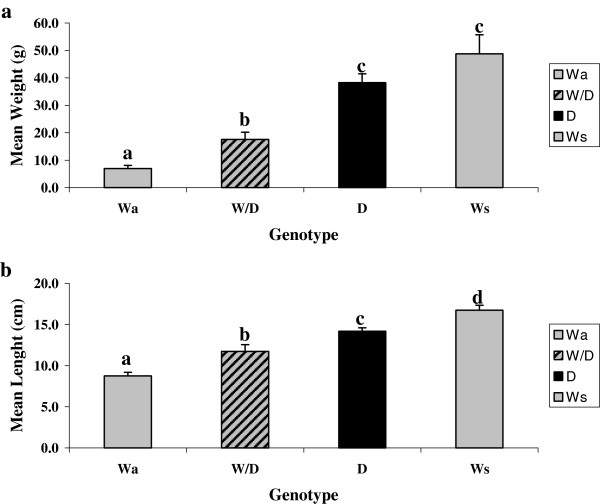
**Fish weights (a) and lengths (b).** Weights and lengths are shown for age-matched wild (Wa), size-matched wild (Ws), domesticated (D) and wild-domesticated hybrid progeny rainbow trout used in this study. Statistically distinct groups are indicated by different letters over the bars.

For analysis, six fish were selected from each of the groups (Wa, Ws, D and W/D) during February 2008. At this time, the domesticated strain individuals had grown to a body size that closely matched that of the 2006 W strain fish (Figure 1). Liver tissue was sampled from six individual fish from each experimental group; Wa, Ws, D and W/D rainbow trout. Lengths and weights (Figure 1) at the time of sampling varied between all groups. In terms of fish weight, domesticated and size-matched wilds were the largest in terms of mass relative to wild-domesticated hybrid and age-matched wild groups. Prior to tissue sampling no food was provided to the fish for a 24 hr period. Fish were terminated by over-anaesthetization with 200 mg/L tricaine methane sulfonate plus 400 mg/L sodium bicarbonate (1:2 ratio), followed by decapitation.

### RNA extraction

Liver tissue was excised and placed immediately in RNAlater as specified by the manufacturer (Ambion) and stored at -80°C until analysis. Total RNA was extracted from individual liver tissues using TRIzol reagent (Invitogen) by pellet pestle homogenization (Kontes) and purified using Qiagen’s RNeasy MinElute Cleanup kit. Quality and quantity of RNA was examined spectrophotometrically at 260 nm and 280 nm and RNA purity estimated using 260/280 ratio ≥ 1.8.

### Microarray hybridization

Microarray experiments were designed to comply with Minimum Information About a Microarray Experiment (MIAME) guidelines [43] and all scanned images and quantified raw data files have been deposited in Gene Expression Omnibus [44] with GEO platform accession number [GSE45151]. All hybridizations were carried out using 44K salmonid oligonucleotide arrays containing four arrays per slide, provided by the consortium for Genomics Research on All Salmon Project (cGRASP) [45,46]. A reference design was used in all hybridizations, composed of pooled total RNA from wild size-matched rainbow trout (n = 6). This pooled sample served as the Cy3 labelled control (see below) to which each individual fish was compared. Each experimental group (D, W/D, Wa and Ws) included 6 individual fish. Thus, in total 24 44K oligo-arrays were utilised during this study. Total liver RNA for individual fish from each experimental group was hybridized to one of four arrays per individual slide. For each experimental group the positioning on the slide was: D, W/D, Wa and Ws.

Total RNA (100 ng) for both reference and experimental samples was converted to cDNA and cRNAs derived therefrom were labelled with the appropriate fluorescent dye using the Agilent Low Input Quick Amp (LIQA) kit, following manufacturer’s instruction. Agilent Spike A control and Spike B control were added to reference (Cy3) and experimental (Cy5) samples, respectively. Following denaturation (65°C for 10 min) and cRNA synthesis (40°C for 2 hr) steps, the reaction was heated at 70°C for 15 min and, then placed directly on ice to inactivate the AffinityScript enzyme. cRNA was then labelled with the appropriate fluorescent dye by the addition of 6 μL of Transcription Master Mix Cocktail containing either Cy3 or Cy5. The reaction mix was incubated at 40°C for 2 hr and, subsequently stored at -80°C until use. Labelled cRNA samples were thawed and then purified using Qiagen’s RNeasy mini spin columns. Labelled cRNA samples were quantified spectrophotometrically at 260 nm, incorporation of Cy dyes and specific activity of each sample were calculated as per manufacture’s guidelines (Agilent). Hybridizations were carried out in accordance with LIQA kit protocol, using 825 ng of experimental and reference pool cRNA. 100 μL of this hybridization reaction mix was loaded in order D, W/D, Wa and Ws to each of the respective individual grids on the 44K oligo-array using Agilent’s SureHyb enabled Hybridization reaction chamber and gasket slides. Hybridization was carried out at 65°C for 17 hr in a rotary hybridization oven at 10 rpm. Following hybridization, slides were washed as per manufacture’s protocol. Slides were scanned immediately using ScanArray Express (PerkinElmer, Waltham, MA, USA) scanner at 5 μm resolution at 90% laser power, with PMT values of 85 for Cy3 channel and 70 for Cy5 channel.

### Data analysis

Scanned arrays were quantified using Imagene 9.0 (BioDiscovery, EL Segundo, CA, USA) and data files processed prior to import into GeneSpring 12.0 (Agilent Technologies, Inc, Santa Clara, CA, USA). Data was imported into GeneSpring under the following conditions: raw data was converted to a threshold value of 1.0, raw data was log_2_ transformed, and LOWESS normalisation and baseline transformation to the median value of all samples was performed. As a quality control, data for each slide was examined using scatter plot and MA-plot to examine dye bias and technical variation between slides for each biological replicate.

**Figure 2:**
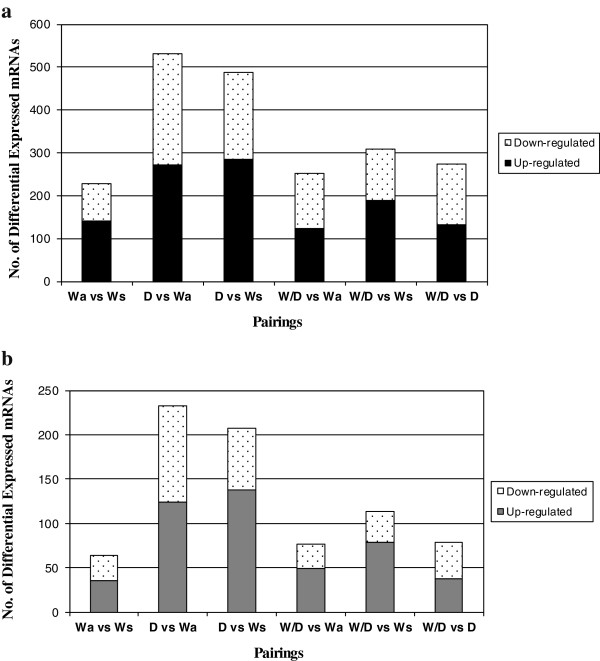
**Significant differences found in mRNA levels between group pairings. a)** Significant differences as determine by ANOVA *P* ≤ 0.05. **b)** Significant differences as determine by ANOVA *P* ≤ 0.05 with fold change of ≥ 2. Each bar chart shows the amount of up-regulated and down-regulated mRNAs found in each group pairing of fast-growing domesticated (D), slow-growing age-matched wild (Wa), slow-growing size-matched wild (Ws), and first generation hybrid cross (W/D) rainbow trout.

Of the 43,689 entities (oligos spotted on the array), 9,386 were present in this study and this list was used for the final statistical tests. This entity list was created by filtering based on expression (lower intensity cut-off ≤ 300) and flags to account for background, false positives and absent spots. Statistical analysis was performed using a one-way ANOVA assuming equal variance with Tukey’s post-hoc and Benjamini Hochberg (FDR) multiple corrections. Significance was determined using *P* values ≤ 0.05 or ≤ 0.01. Figure 2 details the number and type of expression found to differ between all pairwise comparisons of Wa, Ws, D and W/D-hybrid, and a list of annotated entities can be found in the additional files provided (Additional files 1, 2, 3, 4, 5, 6).

Hierarchical clustering was performed on all mRNAs with signals above background on the array and on significant mRNAs found during analysis, using Euclidian distance measurement and clustering based on condition (genotype) and mRNA level (GeneSpring). It should be noted however that hierarchical clustering in reference to its use here is not a statistical analysis method, and in this case was only used as a means of visually inferring relationships among genotypes. As an additional quality control, a 3D-PCA plot was examined to assess group cohesiveness (within and between) each of the genotypes (Wa, Ws, D and W/D). Clustering according to genotype was evident, with all individuals within a genotype clustering together. Some overlapping of individuals within the D and W/D groupings was noted. Following statistical analysis of differentially expressed mRNAs, all replicates were removed from mRNA entity lists. For replicate removal of same named mRNAs, in each case the first mRNA within each replicate group was selected. This was done consistently for removal of same name replicates from each entity list. Venn diagrams were employed during analysis to determine the proportion of mRNAs that were mutually expressed in comparisons of D to wild-type groups and those unique to Wa or Ws groups relative to D groups. This was performed in order to draw some conclusions with regards the relevance and or necessity for size matching fish during experimental design. Venn diagram breakdown was also used in this study to examine mutually expressed mRNAs found on comparison of wild-domesticated hybrid trout in relation to all other groups.

Examination of mRNA regulation patterns between genotypes was performed using SPSS software version 18.0 (IBM). One-way ANOVA assuming equal variance with Tukey’s post-hoc and Benjamini Hochberg (FDR) multiple corrections was performed on a combined list of mRNAs found to be significantly different for pairings of D to wild-type trout during microarray statistical testing (*P* ≤ 0.05 and fold change ≥ 2). Statistical testing was performed on normalized log expression values from all individual fish within the D, W/D and Wa and Ws groups and differences were noted as significant with a *P* value ≤ 0.05. Regulation of mRNAs was defined as additive if significant differences were noted for comparisons of both W/D to W and W/D to D. Regulation of mRNAs was defined as non-additive if the W/D-hybrid was significantly different from one but not the other parental strains. Regulation of mRNAs was defined as D-dominant (in reference to D) if W/D was significantly different from the wild-type but not from the D parental strain. Regulation of mRNAs was defined as D-recessive (in reference to D) if W/D was significantly different from D but not the W parental strain. During analysis, there were some cases where a mRNA resembled additive effect but was not significantly different from both parents. To confirm an additive effect or no effect (Ne) of genotype for these mRNAs linear regression analysis was used. Linear regression analysis was performed for each individual mRNA. Expression values (non-log expression values) for an individual mRNA were plotted across the three genotype groups and the slope of the line determined (SPSS version 18.0, IBM). The effect of genotype on mRNA regulation was classified as resembling additive (and included in the additive category) if the slope of the line was significantly different from zero (*P* ≤ 0.05); if the slope was not significantly different from zero, mRNA regulation was classified as no effect (Ne) of genotype.

Functional pathways were assigned where possible to the significant mRNAs found in comparison between all group pairings using UniProt Knowledgebase [47], EMBL-EBI [48], Gene Ontology (GO) information provided in array annotation file and an in-house database.

## Results

### Entity list generation and statistical analysis

Levels of liver mRNAs were analyzed using a 44K oligo-array (consortium for Genomics Research on All Salmon Project; cGRASP) for four groups of rainbow trout (fast-growing domesticated (D), wild-domesticated hybrid (W/D), slow-growing age-matched wild-type (Wa), and slow-growing size-matched wild-type (Ws)). Comparisons among these groups allowed assessment of the influence both of genotype (domesticated vs. wild, and hybrid) and developmental stage (body size and age) on mRNA levels. Following data normalisation and quality control filtering a total of 9,386 out of 43,689 oligos spotted on the array platform (21.5% of those on the array) were found to be present within the confines of this experiment. Significant entity lists were generated for each group pairing and were also further filtered to examine differences greater than 2 fold to focus on mRNAs with major differences in expression. Figure 2 details the number of mRNAs found significant within each pairing, before (Figure 2a) and after (Figure 2b) filtering on fold change, and with replicate same named mRNAs entities removed. Statistical analysis using a one-way ANOVA was also performed with significance levels adjusted to *P* ≤ 0.01 for more stringent analysis (Additional file 7) which in most groups reduced the number of significant mRNAs to nearly half. In total, 733 or 7.8% (same named replicates removed) of all entities expressed on the array were deemed to be significantly different (ANOVA, *P* ≤ 0.05) in terms of mRNA levels among any of the different rainbow trout group pairings (Figure 2a). mRNAs with major changes were defined as those having a greater than 2-fold difference (condition 1/condition 2) in mRNA level between any of the group pairings. This focus decreased the number of mRNAs to 351 (3.7%) of all expressed entities on the array (Figure 2b) and will be referred to as the comprehensive significant entity list.

### Differences in mRNA levels found for W (wild-type), D (Domesticated), and W/D (wild-domesticated hybrid) rainbow trout

Figure 2b details the number of mRNAs found to differ significantly among the various comparisons of D, W/D, Wa, and Ws trout. The largest proportion of mRNAs showing significant differences were noted for D when compared to W-type trout groups (greater than 200 mRNAs), with similar numbers found with comparisons to either age-matched or size-matched wild-type groups. Comparison of the W/D group relative to Wa and to D groups showed similar amounts of differential mRNA expression, whereas comparison of W/D to Ws showed higher amounts of mRNAs with different levels of expression. Some mRNAs (64) were also found to differ between the two wild-type groups (Wa and Ws).

#### Wa relative to Ws trout

**Figure 3:**
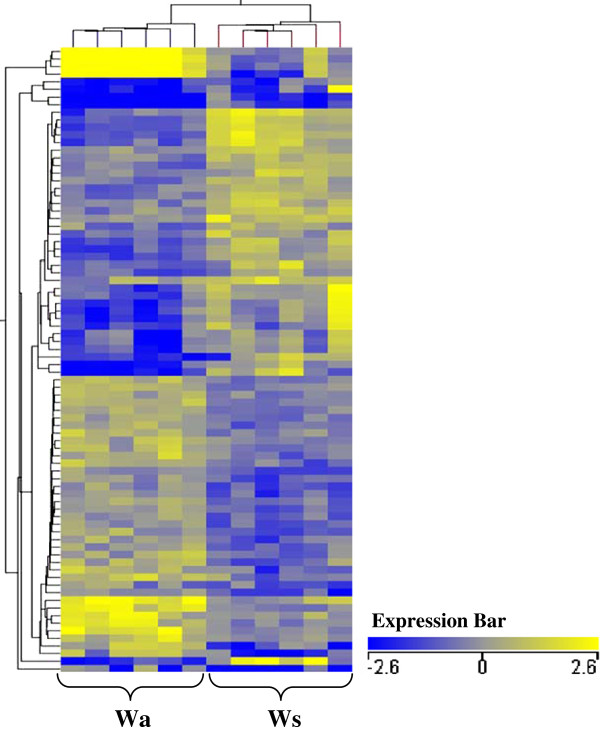
**Hierarchical clustering of mRNA expression profiles in liver tissue for age-matched and size-matched wild-type fish.** Heatmap of hierarchical clustering using Euclidian distance measurement on mRNAs with significantly different expression (*P* ≤ 0.05 and fold change ≥ 2) between Wa relative to Ws groups (individual samples). The X-axis is clustered by group (condition) and the Y-axis by normalised mRNA expression values. Hierarchical clustering of individual fish grouped the fish according to age-matched wilds (Wa) and size-matched wilds (Ws). The expression bar indicates the normalised expression level for each mRNA, which are represented by various colour intensities.

Differences in liver mRNA levels were noted between Wa relative to Ws-type trout (*P* ≤ 0.05). Of the comprehensive significant entity list, 18.2% (64) differed in mRNA expression between Wa relative to Ws groups (Figure 2b). When visualized via heat maps of mRNA levels, individual mRNA expression patterns appeared consistent for all individual fish within each group but differed between the two wild-type groups (Figure 3). Expression profiles within this pairing show similar levels of up or down mRNA regulation (Figure 2b). Note that prior to the application of a fold change ≥ 2 (Figure 2a), Wa differed quite substantially in term of mRNA levels (229 mRNAs) in comparison to Ws-type trout.

#### D relative to Wa and Ws trout groups

**Figure 4:**
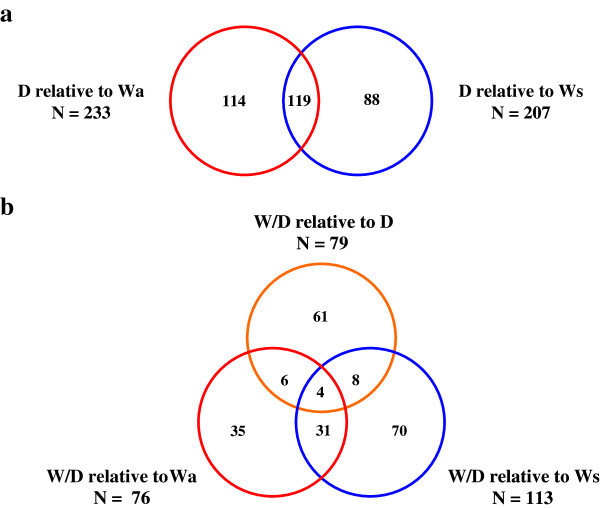
**Venn diagram comparisons of differences in mRNA levels between group pairings. a)** Venn diagram comparison of mRNAs found to differ in expression levels for D relative to Wa and D relative to Ws. The overlapping regions represent common mRNAs found in D relative to both wild-type trout. **b)** Venn diagram comparison of W/D relative to Wa, Ws and D trout. Overlapping regions represent common mRNAs found in all group pairings and between two group pairings.

**Figure 5:**
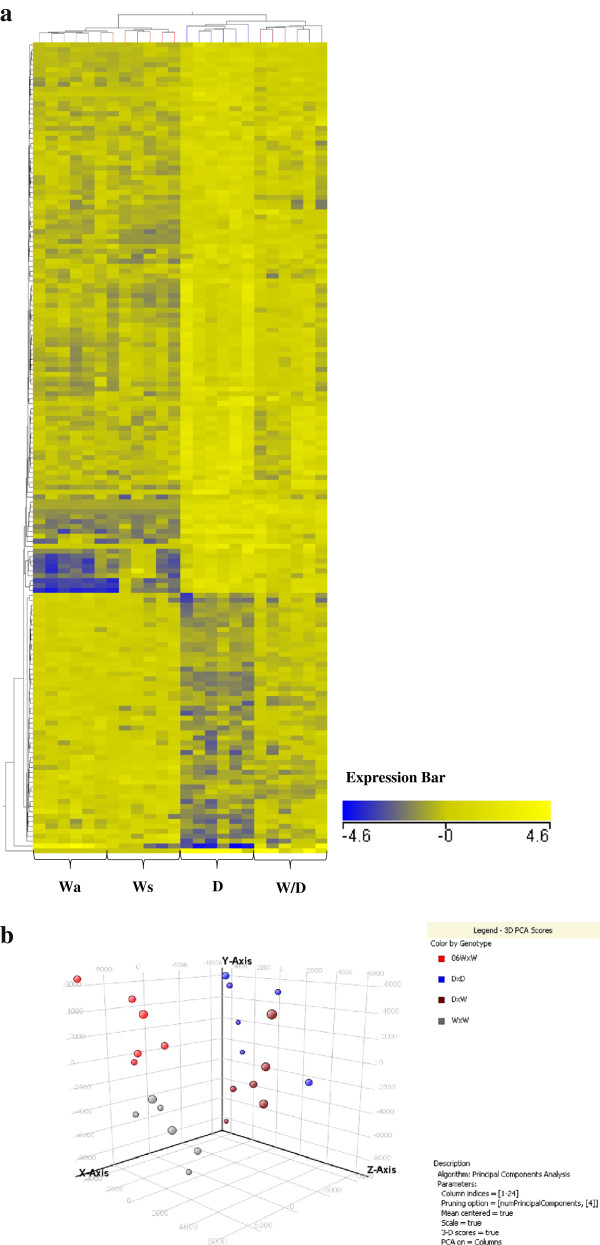
**Hierarchical clustering of mRNA expression profiles in liver tissue of all individual fish, including 3D-PCA plot. a)** hierarchical clustering using Euclidian distance measurement on all mRNAs with significantly different expression (*P* ≤ 0.05 and fold change ≥ 2, 165 mRNAs total, includes replicates) common between D relative to W groups. The X-axis is clustered by group (condition) and the Y-axis by normalised mRNA expression values. Clustering was done using all individual samples for age-matched wilds (Wa), size-matched wilds (Ws), domesticated (D) and wild-domesticated hybrid (W/D). The expression bar indicates the normalised expression level for each mRNA (intensity), which are represented by various colour intensities. **b)** 3D-PCA plot showing variation based on biological variables. Three-dimensional principal component plot with each axis explaining a certain percentage of variation among all samples (n = 24). X-axis: 12.91% Y-axis: 9.47% and Z-axis: 6.47%. Samples are coloured according to genotype assignment with age-matched wild (grey), size-matched wild (red), domesticated (blue) and wild-domesticated hybrid (reddish brown).

The largest proportion of differences in mRNA levels in liver was found in comparisons of D relative to either wild-type group. Of the comprehensive significant entity list, 233 (66.4%) and 207 (58.9%) mRNAs differed significantly between D and Wa and Ws, respectively (Figure 2b). Venn diagram analysis of both significant entity lists showed 119 of these mRNAs were shared in comparisons of D to Wa and to Ws-type trout (Figure 4a). Figure 2a and 2b detail the proportion of up and down mRNA regulation for all significant mRNAs found between pairings of D relative to W-type trout. For D relative to Wa similar levels of up and down mRNA regulation are noted, whereas for D relative to Ws a higher proportion of mRNAs were up-regulated as opposed to down-regulated (Figure 2b). Hierarchical clustering of those mRNAs found common to both wild-type in comparisons to D produced two cluster groups, with domesticated (D) and wild/domesticated (W/D) rainbow trout groups clustering together and, age-matched (Wa) and size-matched (Ws) wild-type rainbow trout clustering together (Figure 5a). Examination of mRNA expression levels (normalised expression value and fold change, see Additional files 1 and 2) and type (up or down regulation) were found to be very similar for both wild-type groups relative to D (Figure 5a). However, the unique differences in mRNA levels found here in comparison of D relative to Wa and D relative to Ws (Figure 4a) further emphasises the difference between wild reference groups. Individual variation (n = 24) within and between groups was examined using a 3D-PCA plot (Figure 5b). All samples within each genotype were found to cluster together within the PCA plot, with some overlap seen for D and W/D hybrids as expected.

#### W/D hybrid relative to D and wild-type trout

W/D hybrid group were compared to Wa, Ws and D groups. The largest degree of variation in mRNA levels was found in comparison of W/D with Ws, with 113 mRNAs (32.2%) from the comprehensive significant entity list differing in this pairing. Similar proportions of differences in mRNA levels were found in comparison of W/D relative Wa and D groups (76 or 21.6%, and 79 or 22.5%, respectively; Figure 2b). A greater proportion of mRNAs were up-regulated in W/D relative to W-type fish, while similar levels of up/down regulation were noted for those mRNAs significant in W/D relative to D fish (Figure 2b). Venn diagram analysis of significant entity lists for each W/D pairing, show very few (n = 4) mRNAs to be common between all groups (Figure 4b). However, 31 mRNAs were shared between pairings of W/D to Wa and Ws-type trout (Figure 4b).

### Relationship of genotype and mRNA levels

**Figure 6:**
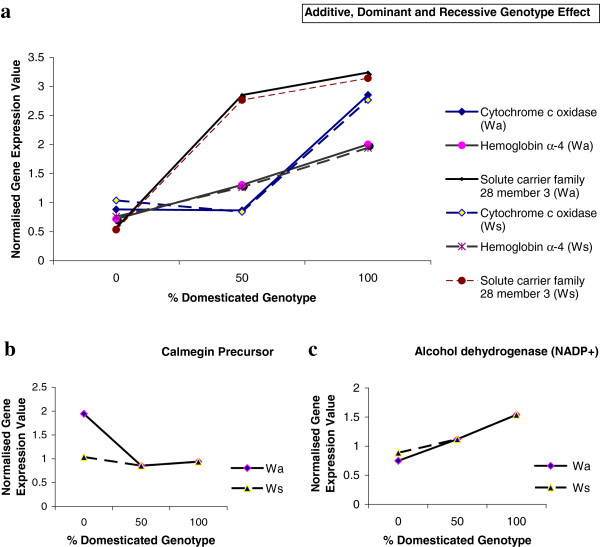
**Representative mRNAs showing the influence of domesticated genotype on normalised gene expression.** Strains are pure wild (0%), wild-domesticated hybrids (50%) and pure domesticated (100%). Expression values for both Wa (solid line) and Ws (dashed line) are shown. **a)** illustrates examples of additive (hemoglobin subunit alpha-4), D-dominant (solute carrier family 28 member 3) and D-recessive (cytochrome C oxidase subunit 4 isoform 2) genotypic effect. Data for Ws fish in 6a, (only) has been shifted (3%) for clarity. **b)** illustrates the effect of fish stage and gives an example of a mRNA that showed D-dominant (Wa) or no effect (Ws) of domestication depending on whether Wa or Ws is considered, respectively. **c)** gives an example of a mRNA that showed no significant difference of hybrid from both parental strains but when analysed using regression analysis was classified as resembling additive effect. The standard error of the mean for this data ranged from 0.004 - 0.564.

Those mRNAs (321) found to be statistically different (*P* ≤ 0.05 and fold change ≥ 2) between D relative to W-type rainbow trout during microarray analysis were used to determine the influence of genotype (domesticated vs. wild, and hybrid) and developmental stage on mRNA regulation. One-way ANOVA was perform on normalised log intensity values for D, W-type and W/D hybrid groups, to determine additive (a), D-recessive (r), or D-dominant (d) genotype effects for the 321 mRNAs (Table 1). Examples of each genotype effect are shown in Figure 6a. This list of differentially expressed mRNAs between D relative to W-type can be divided into three groups. Group A represents differentially expressed mRNAs found between D and both wild-type groups, group B represents mRNAs that differed significantly only between D relative to Wa type trout, and group C represent mRNAs that differed significantly only between D relative to Ws type trout. For group A, similar levels of additive, D-dominant and D-recessive genotype control were noted, however a tendency for increased prevalence of additive and D-recessive model can be seen (Table 1). Within this group it was found that a D-dominant genetic variation was more prominent for up-regulated as opposed to down-regulated mRNAs (data not shown). Table 1 shows a large proportion of mRNAs within group A that were regulated in a D-recessive or D-dominant manner relative to domesticated fish. These results indicate a strong influence of both the wild and the domesticated genome in mRNA regulation. High levels of concordance (84% of all mRNAs within group A), for additive, D-recessive and D-dominant effects, was found when using either Wa or Ws type trout for comparisons. This implies that for group A, mRNA regulation is most likely due to the effect of genotype and not a result of age or stage differences between D and wild fish.

**Table 1:** Relationship between genotype and mRNA levels

**Genotype effect**				
*Group A: Shared differences in mRNA D relative to W-type*				
	***r***	***d***	a*	***Ne***
Wa	40	26	48	1
Ws	44	32	36	0
Concordance	36	24	32	0
*Group B: Unique difference in mRNA D relative to age-matched Wilds*				
Wa	39	41	27	0
Ws	37	3	32	36
Concordance	32	3	13	0
*Group C: Unique differences in mRNA D relative to size-matched Wilds*				
Wa	11	7	37	30
Ws	10	54	20	0
Concordance	10	7	12	0

Table shows the number of mRNAs from the D relative to W-type entity list that showed additive (a), D-recessive (r) or D-dominant (d) mRNA regulation patterns relative to the domesticated parent. Included are those mRNAs that were found to differ significantly in D relative to Wa only and D relative to Ws only. Included in the table are those mRNAs that showed no effect (Ne) of genotype on mRNA regulation.

*a: additive (includes mRNAs that resemble additive effect), r: D-recessive (relative to domesticated), d: D-dominant (relative to domesticated) and Ne: no effect of genotype.

Table 1 shows that for groups B and C the greatest degree of concordance when analysing the effect of genotype (between Wa and Ws groups) on mRNAs abundance was seen for D-recessive regulation. A significant proportion of mRNAs within these groups were unaffected by genotype (Ne). For example, mRNAs that displayed a D-dominant response when comparing W/D to D and Wa groups, showed either no effect (Figure 6b) or were consistent with additive effects when comparing the same mRNA expression for W/D to D and Ws trout groups. These results indicate that the controlling factor for differences in these mRNAs is most likely dictated by differences in fish developmental stage or life history between domesticated and wild rainbow trout rather than by genotype. Examples of additive (a), D-recessive (r) and D-dominant (d) genotype effects are shown in Figure 6a, along with illustrations of the effect of development stage on expression levels (Figure 6b) and those resembling additive (Figure 6c).

### Physiological differences in liver for Wa, Ws, D and W/D rainbow trout

In order to determine which physiological pathways differ between the domesticated and wild-type strains examined, functional pathways were assigned where possible to the comprehensive significant entity list (351 mRNAs) identified from all group comparisons. The functional pathways were categorised under 16 umbrella terms (see Additional files 1, 2, 3, 4, 5, 6). Physiological pathways which showed alterations between groups were primarily associated with the following groups: response to stimulus (including stress/immune response and oxidation-reduction), cell/tissue structure and development (including cell adhesion, muscle and cytoskeletal development), and transport (primarily in oxygen and metabolite transport).

**Figure 7:**
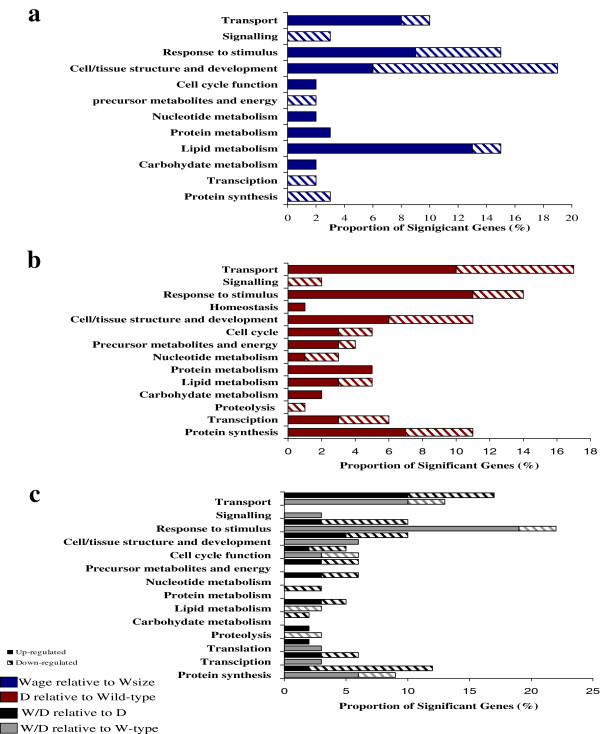
**Physiological pathways found significant in pairings of Wild-type (W), domesticated (D) and wild-domesticated hybrid (W/D) populations. a)** the physiological pathways up- or down-regulated in Wa relative to Ws (64 mRNAs total). **b)** the physiological pathways up- or down-regulated D relative to both W-type fish (119 mRNAs total). **c)** physiological pathways up- or down-regulated in W/D relative to W-types (only common, 31 mRNAs) and D (79 mRNAs total) groups.

Differences in physiological processes between Wa and Ws type trout groups were primarily in cell/tissue structure and development, response to stimulus, lipid metabolism, and transport (Figure 7a). In terms of expression profiles, it was noted that the majority of mRNAs which showed up-regulation in Wa relative to Ws trout were associated with metabolic pathways, mainly lipid metabolism, transport systems and response to stimulus (oxidation-reduction reactions). Down-regulated mRNAs within this pairing related mostly to cell/tissue structure and development (skeletal muscle development) and response to stimulus (Figure 7a).

The main physiological pathways which differed between D to W-type groups were found in transport, response to stimulus, cell/tissue structure and development, metabolism (combined metabolic pathways) and protein synthesis (in Figure 7b only mRNAs commonly affected in Wa and Ws are shown). Of the mRNAs within this group, 15% had unknown functions and therefore could not be assigned to any physiological pathway group. For mRNAs which demonstrated unique differences in mRNA levels in D relative to Wa or D relative to Ws, the proportion of mRNAs and types of physiological pathways were similar for both groups, but the specific mRNAs and mRNA expression profiles (up/down regulation) differed (see Additional file 8). Some physiological pathways which differed in D relative to Wa, but not Ws, were homeostasis, protein metabolism, translation and apoptosis.

Figure 7c details the physiological pathways which differed between W/D hybrid relative to W-type and D-type trout (only shared mRNAs are shown for both W-types). Although few mRNAs were shared between W-type trout and D when either was compared to W/D (Figure 4b), some similarities were noted in the types of altered physiological pathways. While a large proportion of mRNAs within these pairings were unknowns, the main pathways identified were transport, response to stimulus, cell/tissue structure and development and protein synthesis. Difference in physiological pathways for W/D relative to D, in relation to W/D relative to W-type, were in signalling, generation of precursor metabolites and energy, nucleotide, protein and carbohydrate metabolism. Figure 4b details the number of mRNAs that differ in expression levels for W/D relative to Wa only and Ws only.

## Discussion

To assess the genetic and physiological transformations that occur as part of the domestication process, the present study measured differences in mRNA levels between slow-growing wild and fast-growing domesticated strains of rainbow trout, and analyzed the influence of combining domesticated and wild genomes in F_1_ wild-domesticated hybrid progeny. This research also assessed the effect of comparing mRNA levels of domesticated and hybrid genotypes to two different wild-type control groups that were either size-matched or age-matched to the domesticated genotype.

Substantial significant differences in mRNA expression were found for fast-growing domesticated rainbow trout relative to the slow-growing wild trout, with 5.4% of all detected mRNAs differentially expressed between these genotypes. Further, significant differences were also observed for both of these parental strains relative to their wild-domesticated hybrid F_1_ progeny, although to a lesser extent than between parental groups. Among all detected mRNAs, hybrids possessed 3.3% and 2.7% of mRNAs with different expression levels relative to size-matched and age-matched wild trout (respectively), and 2.9% of mRNAs differed when compared to domesticated trout. The lowest proportion of differentially expressed mRNAs was noted in comparison of age-matched and size-matched wild rainbow trout, with 2.4% of all detected mRNAs differing in this pairing. A related study comparing wild rainbow trout and a domesticated strain (different from the strain used in the present study) found similar results, where 6% of all detected mRNA differed in liver tissue [34]. Previous work on brook charr found 4.16% of all detected liver mRNAs differed between domesticated and wild populations at the juvenile stage [39]. Studies with Atlantic salmon whole fry found that 1.4 -1.6% of all detected mRNAs differed in hybrid progeny with respect to their parental populations [37], whereas 6.4% of all detected mRNAs differed in second generation farmed wild backcross relative to wilds [17]. Similar results for hybrid relative to parental populations were observed in a study of normal and dwarf lake whitefish [41]. Slight differences among these data sets likely arise by differences in experimental design (microarray platform, specific tissues vs. whole fry, developmental stages assessed) and variation due to the salmon species under investigation.

Similar proportions of mRNAs are up- vs. down-regulated in domesticated fish relative to their wild counterparts (a slightly higher proportion of up-regulation was seen in the comparison with Ws as opposed to Wa trout). These results are different from the findings of Tymchuk et al. [34] who found a higher representation of down-regulated mRNAs in the liver tissue of domesticated relative to size-matched wild rainbow trout. In the present study, many of the same mRNAs were found to be concordantly regulated in domesticated trout relative both to age-matched and size-matched wild trout. A concordant response is consistent with differences between slow-growing wild and fast-growing domesticated strains being changes that are stable across developmental stages and rearing conditions, and thus may be critical changes that have arisen during the domestication process. It is important to consider that although the domesticated strain used in this study has undergone selection for enhanced growth performance and shows vastly different growth rates to their wild counter parts, not all genetic differences between the strains will have arisen from domestication selection and not all will be related to growth. Other unintentional differences in behaviour, morphology and physiology likely have also arisen and could account for some of the genetic differences noted here. Between wild-domesticated hybrids and parental groups, proportionally more mRNAs were up-regulated relative to age- and size-matched wild trout, whereas similar levels of up and down mRNA regulation were observed relative to domesticated trout.

The analysis of hybrid progeny in conjunction with the pure domesticated and wild parental strains allowed examination of the influence of genotype on mRNA regulation. Specifically, co-dominant expression is occurring if the level of mRNA product in the hybrid is intermediate between the parental strains, D-dominant expression if levels resemble the domesticated strain (equivalent to W-recessive with respect to the wild strain), D-recessive if levels resemble the wild-type strain (equivalent to W-dominant with respect to the wild strain), and over- or under-dominant if levels are respectively greater or lower than all the strains. For those mRNAs that showed concordant responses between domesticated and both wild-type groups (Wa and Ws), hybrid inheritance patterns showed similar levels of additive, D-dominant and D-recessive control. However, a slightly higher proportion of additive and D-recessive modes of regulation can be seen. These findings are concordant with a previous study examining mRNA abundance differences between wild and domesticated strains of Atlantic salmon from two environments [35]. The large proportion of mRNAs showing recessive and dominant genotype regulation suggests specific genetic influences of both the wild and domesticated genome in mRNA regulation in hybrids. The prevalence of dominant and non-additive responses reveals that introgression between domesticated and wild populations considerably alters the genetic control of mRNA levels from that evolved in wild individuals, and therefore may disrupt gene regulatory systems important for developing phenotypes for optimal fitness in nature. In contrast to the responses seen for mRNA levels, previous research examining the influence of genotype on selected traits such as growth and behaviour found mainly additive regulation [5-8,25]. In such cases where F_2_ progeny have been examined, it is anticipated that the backcrossing of F_1_ hybrid genome combination to wild-type would further disrupt mRNA regulatory systems via outbreeding depression. In contrast, numerous studies examining the influence of genotype on mRNA levels have found mainly non-additive, dominant or transgressive modes of regulation [17,36,40,41]. If we consider that the expression of a particular phenotypic trait such as growth rate or maturity are a result of complex networks of genes working in unison, as opposed to a single gene, it is possible that non-additive genotype effects noted in gene regulation responses are masked when gene complexes are formed to give rise to the visible phenotype [32]. Based on the combined results of the current and many previous studies, we agree with the conclusion of Normandeau et al. [36] and Roberge et al. [17] that the consequences of hybridization on both mRNA regulation and phenotype expression are highly dependent on the specific genetic architecture of the crossed populations and therefore, highly unpredictable. The number of fish per genotype (n = 6) used in the present analysis of genotype effects on mRNAs regulation may have generated levels of variance that would prevent identification of differences among the groups for specific genes, and as such, actual differences may be somewhat greater. It is also possible that some of the effects seen may be a result of strain variation within each group which could generate non-homogeneity of genotype among the W/D hybrid fish selected, despite each showing intermediate growth rate and body size relative to the parental strains. Although the issue of within and between genotype variation was examined as part of this study and, little variation between individuals was noted, further study including analysis of additional individuals and strains would be beneficial.

To assess whether specific biological pathways were being influenced, functional assignments for differentially regulated mRNAs were determined for each of the genotypes. Differences between domesticated and wild rainbow trout showed a high representation of mRNAs involved in transport, metabolism, response to stimulus, cell/tissue structure and development, protein synthesis and transcription. The majority of mRNAs involved in these processes had higher mRNA levels in domesticated trout. Examination of alteration in fish physiology for domesticated and growth hormone (GH) transgenic coho [38] and domesticated rainbow trout [34] observed similar changes in stress and immune response, cell and tissue structure, energy production and protein synthesis physiological pathways. Comparable results for alterations in fish physiology have also been seen for brook charr [39] and Atlantic salmon [17,37]. The combination of these results suggests that similar biological pathways are altered in multiple species of domesticated fish, as well as GH transgenic fish, in order to support faster rates of growth, and that these changes may be both causal of or responsive to the underlying genetic variation that has led to altered phenotypes in fast-growing strains [34].

Specifically, in terms of metabolism, energy acquisition and utilisation, mRNAs displaying the highest level of fold change between domesticated and wild strains were Apo-A1-2 precursor, ELOVL FA elongase 6, FAA and protein canopy homolog 2 precursor, NADH-ubiquinonereductase chain 2, cytochrome C and COX IV-2, with under expression of ACBP. The elevated levels of many mRNAs involved in lipid metabolism may reflect the greater demand in domesticated fish for growth-related resources necessary to support increased growth performance [1,40]. Additionally, many mRNAs associated with protein synthesis were over-expressed in domesticated trout. Protein synthesis plays a strong role in fish growth through mediation of many growth related pathways [1,49]. mRNAs which displayed the highest level of over-expression were 40S ribosomal proteins (S5 and S21) and superoxide dismutase. Although superoxide dismutase has been linked to other roles which include antioxidant defence, it is included within this group for its role in protein biosynthetic process and growth regulation. Given that the liver tissue is highly involved in protein turnover, amino acid metabolism and lipid metabolism for its role in protein biosynethic during periods of accelerated growth the elevation of mRNAs within these pathways for the domesticated population was not unexpected.

During this study many mRNAs associated with oxygen and macromolecule transport were found to be expressed at a high level in domesticated relative to wild trout. mRNAs involved in these pathways may show increased levels due to the higher metabolic rate of the domesticated trout, required for increased growth and nutritional absorption. Notable with respect to transport, mRNAs involved in ion transport were found to be down-regulated in domesticated fish. mRNAs which displayed the highest level of over-expression were, solute carrier family 28 member 3, hemoglobin subunits beta (β and β-1,-2) and alpha (α and α-4) and vitellogin-3 precursor. Similar results of increased expression of hemoglobin beta and alpha subunits in liver tissue were reported by Rise et al. [50] for examination of GH transgenic coho expression. Tymchuk et al. [34] observed different results to those described above, with increased expression of hemoglobin found only in the brain tissue of domesticated rainbow trout. Differences seen between studies maybe due to, strain selection, variance in fish growth rate, or, perhaps to differences in the vascular circulatory systems in the liver tissue.

Results of differential mRNAs expression in liver tissue showed over-expression in domesticated trout of many mRNAs involved in the activation and regulation of the complement pathway and innate immunity. Increased expression in domesticated trout was noted for lectin precursor, Complement C3-1, Fucolectin 6 precursor, Complement factor b precursor, C-type lectin domain family 4 member m and decreased expression of primary defence mechanisms exemplified by Ig mu chain C region membrane bound form. These results are contradictory to the findings of Tymchuk et al. [34] who described down-regulation of many mRNAs involved in the stress and immune system, and attributed this fact to tradeoffs incurred by domesticated trout in order to sustain increased growth rates. Debes et al. [35] also noted down-regulation of some immune related mRNAs in domesticated Atlantic salmon. However, up-regulation of CD59 and MHC class II combined with higher levels of lysozyme C transcript was also noted, and is different from the findings of Tymchuk et al. [34]. It was suggested that elevated levels of these mRNAs may relate to domesticated fish displaying a higher resistance towards vibriosis, a common bacterial disease in aquaculture relative to wild populations [51]. It is possible that increased expression of mRNAs involved in the complement pathway may be explained by an increased host defence system in domesticated rainbow trout, an acute phase response prior to sampling, or alternative uses for these mRNAs in regulatory pathways in domesticated trout.

An objective of this study was to investigate changes in mRNA levels in domesticated trout using different wild-type comparators. Whenever two strains with different growth rates (e.g. wild and domesticated) are being compared, they will, at a specific age, naturally have different body sizes if provided with satiating levels of food. Since body size is linked to developmental stage in many fishes, differences in mRNA levels are anticipated between such groups simply due to the fish being developmentally distinct, rather than due directly to genetic differences causal of the domestication phenotype. To date, studies investigating the effect of wild reference group selection have focused mainly on wild populations from different geographical location [36,37], population groups [40], life stages [39,45,52] and environmental treatment [35]. Bougas et al. [40] clearly demonstrated the influence of wild population selection on changes in mRNA expression by assessing two wild populations from different river systems. Further studies by Debes et al. [35] illustrated the effect of wild rearing environment on differential mRNA expression when either wild population was paired to domesticated populations. The present study aimed to extend previous experiments by our group and others by examining differences that occur within a specific strain reared in the same environment, with one wild group a year older than the other. Domesticated populations were matched to wild-type fish of either the same age or the same size (year older) in order to distinguish alterations in mRNA expression that have arisen due to differences in fish developmental stage rather than due to domestication.

Significant differences in mRNA expression were noted for age-matched relative to size-matched wild-type trout, with 0.68% of all detected mRNAs differing greater than 2-fold (2.4% for all differentially detected mRNAs). When domesticated trout were compared to wild-type trout groups, unique differences in mRNA levels were noted. This comparison reveals how developmental stage and/or age, can alone cause differential mRNA levels, independent of effects arising from domestication. Further confirmation of these findings was found upon analysis of the effect of genotype on mRNA regulation comparing parental to hybrid groups. Unique mRNA expression patterns were observed between domesticated and either age-matched or size-matched wild-type trout, consistent with a clear effect of fish development stage on mRNA levels. The biological pathways influenced by these mRNAs in both groups of wild-type trout also differed, primarily with respect to transport, response to stimulus, and, most strongly, cell/tissue structure and development, and lipid metabolism. The manner in which wilds differed in terms of physiology strongly supports the case of difference due to age, development, and life history. These results suggest that caution should be applied in interpreting data where only one control group (age- or size-matched) is selected in experiments comparing fish with different growth rates.

## Conclusion

The present study has shown that considerable differences in genetics and physiology are associated with strains of domesticated and wild-type rainbow trout. Assessment of genotype effects demonstrated mainly additive and D-recessive mRNA regulation in hybrid progeny. To better understand the consequences of hybridization (and effects on phenotype and risks of introgression of domesticated strains into native populations), further study would be beneficial, including assessing additional strains and species, allelic variation among individuals within strains, and second generation hybrid crosses to identify specific quantitative trait loci influencing morphology, physiology (e.g. growth), and mRNA levels (eQTL analysis). The mRNAs within the D-recessive class may be of particular interest in this regard as we predict that these genomic blocks may show highly significant eQTL associations with growth. The present study revealed the importance of assessing the effect of developmental stage on differential mRNA levels between fast-growing domesticated and slow-growing wild-type groups. Differences in mRNA levels (from that seen in domesticated trout) were more prominent for age-matched as opposed to size-matched wild-type controls, suggesting that matching groups by size (e.g. developmental stage) may provide more biologically meaningful data indicative of genetic differences between strains.

### Availability of supporting data

The data set supporting the results of this article is available in Gene Expression Omnibus (GEO) repository [GEO; http://www.ncbi.nlm.nih.gov/geo/] with GEO platform accession number [GSE45151].

## Competing interests

The authors declare that they have no competing interest.

## Authors’ contributions

SLW wrote the manuscript and performed all bioinformatics and statistical analysis. DS produced the British Columbia family crosses and conducted all microarray hybridizations. RHD, RGD and SLW were involved in the design of the study and data interpretation. All authors read and commented on the manuscript.

## Supplementary Material

Additional file 1 **Significant mRNA entity list (including replicate mRNAs) of domesticated relative to age-matched wild-type rainbow trout (*P-*value of** ≤ **0.05 and fold change of** ≥ **2).** Click here for file

Additional file 2 **Significant mRNA entity list (including replicate mRNAs) of domesticated relative to size-matched wild-type rainbow trout (*P*-value of** ≤ **0.05 and fold change of** ≥ **2).** Click here for file

Additional file 3 **Significant mRNA entity list (including replicate mRNAs) of wild-domesticated relative to domesticated rainbow trout (*P*-value of** ≤ **0.05 and fold change of** ≥ **2).** Click here for file

Additional file 4 **Significant mRNA entity list (including replicate mRNAs) of wild-domesticated relative to age-matched wild-type rainbow trout (*P*-value of** ≤ **0.05 and fold change of** ≥ **2).** Click here for file

Additional file 5 **Significant mRNA entity list (including replicate mRNAs) of wild-domesticated relative to size-matched wild-type rainbow trout (*P*-value of** ≤ **0.05 and fold change of** ≥ **2).** Click here for file

Additional file 6 **Significant mRNA entity list (including replicate mRNAs) of age-matched wild relative to size-matched wild-type rainbow trout (*P*-value of** ≤ **0.05 and fold change of** ≥ **2).** Click here for file

Additional file 7: Figure S1 Significant differences found in mRNA levels between group pairings as determine by ANOVA *P* ≤ 0.01 with fold change of ≥ 2. Bar chart shows the amount of up-regulated and down-regulated mRNAs found in each group pairing of fast-growing domesticated (D), slow-growing age-matched wild (Wa), slow-growing size-matched wild (Ws), and first generation hybrid cross (W/D) rainbow trout. Click here for file

Additional file 8: Figure S2 Physiological pathways found significant in pairings of domesticated (D) and wild-domesticated (W/D) relative to either age- (Wa) or size-matched (Ws) trout populations. S2a.) the physiological pathways up- or down-regulated, unique to D relative to Wa (blue) or Ws (red). S2b.) the physiological pathways up- or down-regulated in W/D relative to Wa (blue), Ws (red) and D (grey) populations. Click here for file

## References

[B1] Blier PU Pelletier D Dutil JD Does aerobic capacity set a limit on fish growth rate? Rev Fish Sci 1997 5 323 340 10.1080/10641269709388604

[B2] Weiler U Claus R Schnoebeleen-Combes S Louveau I Influence of age and genotype on endocrine parameters and growth performance: a comparative study in Wild boars, Meishan and Large White boars Livest Prod Sci 1998 54 21 31 10.1016/S0301-6226(97)00165-6

[B3] Diamond J Evolution, consequences and future of plant and animal domestication Nature 2002 418 700 707 10.1038/nature01019 12167878

[B4] Myers JM Hershberger WK Saxton AM Iwamoto RN Estimates of genetic and phenotypic parameters for length and weight of marine net-pen reared coho salmon (Oncorhynchus kisutch Walbaum) Aquacult Res 2001 32 277 285 10.1046/j.1365-2109.2001.00556.x

[B5] Tymchuk WE Devlin RH Growth differences among first and second generation hybrids of domesticated and wild rainbow trout (Oncorhynchus mykiss) Aquaculture 2005 245 295 300 10.1016/j.aquaculture.2004.11.007

[B6] Tymchuk WE Biagi C Withler R Devlin RH Growth and behavioral consequences of introgression of a domesticated aquaculture genotype into a native strain of coho salmon T Am Fish Soc 2006 135 442 455 10.1577/T05-181.1

[B7] Glover KA Otterå H Olsen RE Slinde E Taranger GL Skaala Ø A comparison of farmed, wild and hybrid Atlantic salmon (Salmo salar L.) reared under farming conditions Aquaculture 2009 286 203 210 10.1016/j.aquaculture.2008.09.023

[B8] Solberg MF Skaala O Nilsen F Glover KA Does domestication cause changes in growth reaction norms? A study of farmed, wild and hybrid Atlantic salmon families exposed to environmental stress Plos One 2013 8 e54469 10.1371/journal.pone.0054469 23382901 PMC3561353

[B9] Iwamoto RN Saxton AM Hershberger WK Genetic estimates for length and weight of coho salmon during freshwater rearing J Hered 1982 73 187 191

[B10] Hershberger WK Myers JM Iwamoto RN Mcauley WC Saxton AM Genetic changes in the growth of coho salmon (Oncorhynchus-Kisutch) in marine net-pens, produced by 10 years of selection Aquaculture 1990 85 187 197 10.1016/0044-8486(90)90018-I

[B11] Eimum S Fleming IA Genetic divergence and interactions in the wild among native, farmed and hybrid Atlantic salmon J Fish Biol 1997 50 634 651 10.1111/j.1095-8649.1997.tb01955.x

[B12] McGinnity P Stone C Taggart JB Cooke D Cotter D Hynes R McCamley C Cross T Ferguson A Genetic impact of escaped farmed Atlantic salmon (Salmo salar L.) on native populations: use of DNA profiling to assess freshwater performance of wild, farmed, and hybrid progeny in a natural river environment ICES J Mar Sci 1997 54 998 1008

[B13] McGinnity P Prodöhl P Ó Maoiléidigh N Hynes R Cotter D Baker N O’Hea B Ferguson A Differential lifetime success and performance of native and non-native Atlantic salmon examined under communal natural conditions J Fish Biol 2004 65 173 187 10.1111/j.0022-1112.2004.00557.x

[B14] Fleming IA Hindar K Mjolnerod IB Jonsson B Balstad T Lamberg A Lifetime success and interactions of farm salmon invading a native population Proc Biol sci 2000 267 1517 1523 10.1098/rspb.2000.1173 11007327 PMC1690700

[B15] Skaala O Glover KA Barlaup BT Svasand T Besnier F Hansen MM Borgstrom R Performance of farmed, hybrid, and wild Atlantic salmon (Salmo salar) families in a natural river environment Can J Fish Aquat Sci 2012 69 1994 2006 10.1139/f2012-118

[B16] Hutchings JA Fraser DJ The nature of fisheries- and farming-induced evolution Mol Ecol 2008 17 294 313 10.1111/j.1365-294X.2007.03485.x 17784924

[B17] Roberge C Normandeau E Einum S Guderley H Bernatchez L Genetic consequences of interbreeding between farmed and wild Atlantic salmon: insights from the transcriptome Mol Ecol 2008 17 314 324 10.1111/j.1365-294X.2007.03438.x 18173503

[B18] Lynch M Walsh B Genetics and analysis of quantitative traits 1998 Sunderland, MA: Sinauer Associates

[B19] Ayles GB Baker RF Genetic differences in growth and survival between strains and hybrids of rainbow trout (Salmo-Gairdneri) stocked in aquaculture lakes in the Canadian Prairies Aquaculture 1983 33 269 280 10.1016/0044-8486(83)90407-6

[B20] Dill LM Fraser AHG Risk of predation and the feeding behavior of juvenile coho salmon (Oncorhynchus-Kisutch) Behav Ecol Sociobiol 1984 16 65 71 10.1007/BF00293105

[B21] Johnsson JI Petersson E Jönsson E Björnsson BT Järvi T Domestication and growth hormone alter antipredator behaviour and growth patterns in juvenile brown trout, Salmo trutta Can J Fish Aquat Sci 1996 53 1546 1554 10.1139/f96-090

[B22] Sundstrom LF Lohmus M Johnsson JI Devlin RH Growth hormone transgenic salmon pay for growth potential with increased predation mortality Proc Biol sci 2004 271 Suppl 5 S350 352 15504015 10.1098/rsbl.2004.0189 PMC1810071

[B23] Sundstrom LF Lohmus M Devlin RH Selection on increased intrinsic growth rates in coho salmon, Oncorhynchus kisutch Evolution 2005 59 1560 1569 16153041

[B24] Tymchuk WE Sundstrom LF Devlin RH Growth and survival trade-offs and outbreeding depression in rainbow trout (Oncorhynchus mykiss) Evolution 2007 61 1225 1237 10.1111/j.1558-5646.2007.00102.x 17492973

[B25] McClelland EK Myers JM Hard JJ Park LK Naish KA Two generations of outbreeding in coho salmon (Oncorhynchus kisutch): effects on size and growth CanJ Fish Aquat Sci 2005 62 2538 2547 10.1139/f05-159

[B26] Vandersteen W Biro P Harris L Devlin R Introgression of domesticated alleles into a wild trout genotype and the impact on seasonal survival in natural lakes Evol Appl 2012 5 76 88 10.1111/j.1752-4571.2011.00210.x PMC3353333 25568031

[B27] McGinnity P Prodohl P Ferguson K Hynes R O’Maoileidigh N Baker N Cotter D O’Hea B Cooke D Rogan G Fitness reduction and potential extinction of wild populations of Atlantic salmon, Salmo salar, as a result of interactions with escaped farm salmon P Roy Soc B-Biol Sci 2003 270 2443 2450 10.1098/rspb.2003.2520 PMC1691531 14667333

[B28] Cui X Affourtit J Shockley KR Woo Y Churchill GA Inheritance patterns of transcript levels in F1 hybrid mice Genetics 2006 174 627 637 10.1534/genetics.106.060251 16888332 PMC1602077

[B29] Rottscheidt R Harr B Extensive additivity of gene expression differentiates subspecies of the house mouse Genetics 2007 177 1553 1567 10.1534/genetics.107.076190 17947435 PMC2147974

[B30] Reiland J Noor MAF Little qualitative RNA misexpression in sterile male F(I) hybrids of Drosophila pseudoobscura and D. persimilis BMC Evol Biol 2002 2 16 10.1186/1471-2148-2-16 12223116 PMC126258

[B31] Michalak P Noor MA Genome-wide patterns of expression in Drosophila pure species and hybrid males Mol Biol Evol 2003 20 1070 1076 10.1093/molbev/msg119 12777520

[B32] Gibson G Riley-Berger R Harshman L Kopp A Vacha S Nuzhdin S Wayne M Extensive sex-specific nonadditivity of gene expression in Drosophila melanogaster Genetics 2004 167 1791 1799 10.1534/genetics.104.026583 15342517 PMC1471026

[B33] Hughes KA Ayroles JF Reedy MM Drnevich JM Rowe KC Ruedi EA Caceres CE Paige KN Segregating variation in the transcriptome: cis regulation and additivity of effects Genetics 2006 173 1347 1355 10.1534/genetics.105.051474 16624921 PMC1526654

[B34] Tymchuk W Sakhrani D Devlin R Domestication causes large-scale effects on gene expression in rainbow trout: analysis of muscle, liver and brain transcriptomes Gen Comp Endocrinol 2009 164 175 183 10.1016/j.ygcen.2009.05.015 19481085

[B35] Debes PV Normandeau E Fraser DJ Bernatchez L Hutchings JA Differences in transcription levels among wild, domesticated, and hybrid Atlantic salmon (Salmo salar) from two environments Mol Ecol 2012 21 2574 2587 10.1111/j.1365-294X.2012.05567.x 22519555

[B36] Normandeau E Hutchings JA Fraser DJ Bernatchez L Population-specific gene expression responses to hybridization between farm and wild Atlantic salmon Evol Appl 2009 2 489 503 10.1111/j.1752-4571.2009.00074.x PMC3352448 25567894

[B37] Roberge C Einum S Guderley H Bernatchez L Rapid parallel evolutionary changes of gene transcription profiles in farmed Atlantic salmon Mol Ecol 2006 15 9 20 16367826 10.1111/j.1365-294X.2005.02807.x

[B38] Devlin RH Sakhrani D Tymchuk WE Rise ML Goh B Domestication and growth hormone transgenesis cause similar changes in gene expression in coho salmon (Oncorhynchus kisutch) Proc Natl Acad Sci U S A 2009 106 3047 3052 10.1073/pnas.0809798106 19223591 PMC2651260

[B39] Sauvage C Derome N Normandeau E St-Cyr J Audet C Bernatchez L Fast transcriptional responses to domestication in the brook charr Salvelinus fontinalis Genetics 2010 185 105 112 10.1534/genetics.110.115071 20194962 PMC2870946

[B40] Bougas B Granier S Audet C Bernatchez L The transcriptional landscape of cross-specific hybrids and its possible link with growth in brook charr (Salvelinus fontinalis Mitchill) Genetics 2010 186 97 107 10.1534/genetics.110.118158 20551437 PMC2940315

[B41] Renaut S Nolte AW Bernatchez L Gene expression divergence and hybrid misexpression between lake whitefish species pairs (Coregonus spp. Salmonidae) Mol Biol Evol 2009 26 925 936 10.1093/molbev/msp017 19174479

[B42] Devlin RH Biagi CA Yesaki TY Smailus DE Byatt JC Growth of domesticated transgenic fish - A growth-hormone transgene boosts the size of wild but not domesticated trout Nature 2001 409 781 782 10.1038/35057314 11236982

[B43] Brazma A Hingamp P Quackenbush J Sherlock G Spellman P Stoeckert C Aach J Ansorge W Ball CA Causton HC Minimum information about a microarray experiment (MIAME)-toward standards for microarray data Nat Genet 2001 29 365 371 10.1038/ng1201-365 11726920

[B44] Gene Expression Omnibushttp://www.ncbi.nlm.nih.gov/geo/

[B45] Jantzen SG Sanderson DS von Schalburg KR Yasuike M Marass F Koop BF A 44K microarray dataset of the changing transcriptome in developing Atlantic salmon (Salmo salar L.) BMC Res Notes 2011 4 88 10.1186/1756-0500-4-88 21447175 PMC3073910

[B46] Genomic Research on All Salmon Projecthttp://web.uvic.ca/grasp/microarray/array.html

[B47] UniProt Knowledgebasehttp://www.uniprot.org

[B48] EMBL-European Bioinformatics Institute http://www.ebi.ac.uk/QuickGO/

[B49] Leggatt RA Raven PA Mommsen TP Sakhrani D Higgs D Devlin RH Growth hormone transgenesis influences carbohydrate, lipid and protein metabolism capacity for energy production in coho salmon (Oncorhynchus kisutch) Comp Biochem Phys B 2009 154 121 133 10.1016/j.cbpb.2009.05.010 19470409

[B50] Rise ML Douglas SE Sakhrani D Williams J Ewart KV Rise M Davidson WS Koop BF Devlin RH Multiple microarray platforms utilized for hepatic gene expression profiling of GH transgenic coho salmon with and without ration restriction J Mol Endocrinol 2006 37 259 282 10.1677/jme.1.02031 17032744

[B51] Lawlor JL Dacanay A Hutchings JA Brown LL Sperker SA Differences in pathogen resistance within and among cultured, conservation-dependent, and endangered populations of Atlantic salmon, Salmo salar L Environ Biol Fishes 2008 84 69 78

[B52] Sutherland BJ Jantzen SG Sanderson DS Koop BF Jones SR Differentiating size-dependent responses of juvenile pink salmon (Oncorhynchus gorbuscha) to sea lice (Lepeophtheirus salmonis) infections Comp Biochem Physiol D 2011 6 213 223 10.1016/j.cbd.2011.04.001 21543273

