# Supplementary material for: Influence of developmental stage and genotype on liver mRNA levels among wild, domesticated, and hybrid rainbow trout (Oncorhynchus mykiss)
Source: BMC Genomics. 2013 Oct 2;14:673. doi: 10.1186/1471-2164-14-673 (PMC3851433; doi:10.1186/1471-2164-14-673)
Supplement: Additional file 7: Figure S1 — Significant differences found in mRNA levels between group pairings as determine by ANOVA P ≤ 0.01 with fold change of ≥ 2. Bar chart shows the amount of up-regulated and down-regulated mRNAs found in each group pairing of fast-growing domesticated (D), slow-growing age-matched wild (Wa), slow-growing size-matched wild (Ws), and first generation hybrid cross (W/D) rainbow trout. [file 1471-2164-14-673-S7.doc]

**Figure S1:** Significant differences found in mRNA levels between group pairings as determine by ANOVA P ≤ 0.01 with fold change of ≥ 2. Each bar chart shows the amount of up-regulated and down-regulated mRNAs found in each group pairing of fast-growing domesticated (D), slow-growing age-matched wild (Wa), slow growing size-matched wild (Ws), and first generation hybrid cross (W/D) rainbow trout.
