# Supplementary material for: Influence of developmental stage and genotype on liver mRNA levels among wild, domesticated, and hybrid rainbow trout (Oncorhynchus mykiss)
Source: BMC Genomics. 2013 Oct 2;14:673. doi: 10.1186/1471-2164-14-673 (PMC3851433; doi:10.1186/1471-2164-14-673)
Supplement: Additional file 8: Figure S2 — Physiological pathways found significant in pairings of domesticated (D) and wild-domesticated (W/D) relative to either age- (Wa) or size-matched (Ws) trout populations. S2a.) the physiological pathways up- or down-regulated, unique to D relative to Wa (blue) or Ws (red). S2b.) the physiological pathways up- or down-regulated in W/D relative to Wa (blue), Ws (red) and D (grey) populations. [file 1471-2164-14-673-S8.doc]

**A.**

**b.**

**Figure S2:** Physiological pathways found significant in pairings of domesticated (D) and wild-domesticated (W/D) relative to either age (Wa) or size matched (Ws) trout populations. S2a, the physiological pathways up or down regulated, unique to D relative to Wa (blue) or Ws (red). S2b, the physiological pathways up or down regulated in W/D relative to Wa (blue), Ws (red) and D (grey) populations.
